# The transcriptional programme of *Salmonella enterica *serovar Typhimurium reveals a key role for tryptophan metabolism in biofilms

**DOI:** 10.1186/1471-2164-10-599

**Published:** 2009-12-11

**Authors:** Shea Hamilton, Roy JM Bongaerts, Francis Mulholland, Brett Cochrane, Jonathan Porter, Sacha Lucchini, Hilary M Lappin-Scott, Jay CD Hinton

**Affiliations:** 1 Institute of Food Research, Norwich Research Park, Colney, Norwich, NR4 7UA, UK; 2 Department of Biological Sciences, University of Exeter, Exeter, EX4 4PS, UK; 3 National Laboratory Service, Starcross Laboratory, Staplake Mount, Starcross, EX6 8PE, UK; 4 School of Biological Sciences, University of Southampton, Southampton, SO16 7PX, UK; 5 Department of Microbiology, School of Genetics & Microbiology, Moyne Institute of Preventive Medicine, Trinity College, Dublin 2, Ireland; 6 Shea Hamilton, Faculty of Medicine, Imperial College London, Norfolk Place, London, W2 1PG, UK; Brett Cochrane, Unilever SEAC, Colworth Science Park, Sharnbrook, Bedfordshire, MK44 1LQ, UK

## Abstract

**Background:**

Biofilm formation enhances the capacity of pathogenic *Salmonella *bacteria to survive stresses that are commonly encountered within food processing and during host infection. The persistence of *Salmonella *within the food chain has become a major health concern, as biofilms can serve as a reservoir for the contamination of food products. While the molecular mechanisms required for the survival of bacteria on surfaces are not fully understood, transcriptional studies of other bacteria have demonstrated that biofilm growth triggers the expression of specific sets of genes, compared with planktonic cells. Until now, most gene expression studies of *Salmonella *have focused on the effect of infection-relevant stressors on virulence or the comparison of mutant and wild-type bacteria. However little is known about the physiological responses taking place inside a *Salmonella *biofilm.

**Results:**

We have determined the transcriptomic and proteomic profiles of biofilms of *Salmonella enterica *serovar Typhimurium. We discovered that 124 detectable proteins were differentially expressed in the biofilm compared with planktonic cells, and that 10% of the *S*. Typhimurium genome (433 genes) showed a 2-fold or more change in the biofilm compared with planktonic cells. The genes that were significantly up-regulated implicated certain cellular processes in biofilm development including amino acid metabolism, cell motility, global regulation and tolerance to stress. We found that the most highly down-regulated genes in the biofilm were located on *Salmonella *Pathogenicity Island 2 (SPI2), and that a functional SPI2 secretion system regulator (*ssrA*) was required for *S*. Typhimurium biofilm formation. We identified STM0341 as a gene of unknown function that was needed for biofilm growth. Genes involved in tryptophan (*trp*) biosynthesis and transport were up-regulated in the biofilm. Deletion of *trpE *led to decreased bacterial attachment and this biofilm defect was restored by exogenous tryptophan or indole.

**Conclusions:**

Biofilm growth of *S*. Typhimurium causes distinct changes in gene and protein expression. Our results show that aromatic amino acids make an important contribution to biofilm formation and reveal a link between SPI2 expression and surface-associated growth in *S*. Typhimurium.

## Background

The ability to survive and overcome stress allows non-typhoidal *Salmonella *pathogens to be isolated from a diverse range of environments. Specific serovars of *S. enterica*, including *Salmonella enterica *serovar Typhimurium, are of particular concern to medicine and industry because they cause a significant proportion of foodborne disease worldwide. There has been a controversial suggestion that infection by *Salmonella *may subsequently cause an increase in mortality for up to one year [1], and it is clear that we need to improve our understanding of the behaviour of this pathogen.

Experiments that tracked the spread of *Salmonella *from the farm and within food processing facilities have provided a direct link between bacterial biofilms and the contamination of the resulting food products [2-4]. Surface-associated growth, termed biofilm growth, has been shown to promote the survival of *Salmonella *when exposed to limited nutrient availability, heat, acidic pH, low temperatures and antimicrobials [5-9]. *Salmonella *cells attach and grow on a variety of abiotic and biotic surfaces, and remain viable for many weeks. Indeed, the number of outbreaks of salmonellosis caused by microbial growth on the surfaces of raw fruits and vegetables has increased dramatically in recent years [10-13]. This type of persistence of *Salmonella *in the food chain has become a major health concern, because the detachment of viable cells from a biofilm can cause subsequent contamination of foods during processing.

The profound consequences of biofilm formation in both nature and disease have led to increased efforts to define the characteristics that make biofilm cells physiologically distinct from planktonic (freely suspended) cells, and to identify the properties of microorganisms during surface-associated growth. Cells within a biofilm are heterogeneous, can grow at different rates, and resist antimicrobial treatments [14-16]. Additionally, clusters of biofilm cells are typically encased in a bacterial-derived extracellular matrix that is thought to provide adhesion and strength, as well as act as a physical barrier against the diffusion of antimicrobials [17,18]. *S*. Typhimurium biofilms can form on abiotic surfaces (e.g. glass, polystyrene, stainless steel) and biotic surfaces (e.g. human epithelial cells or gallstones); in many strains, the bacterial cells are associated with an extracellular matrix composed of curli (thin aggregative fimbriae) and cellulose [19-24]. Recent work has shown that strains exhibiting the rdar (red, dry and rough) morphotype, expressing curli and cellulose, are involved in colonisation but do not contribute to the persistence of *Salmonella *on food processing surfaces [25]. *S*. Typhimurium also displays a swarming phenotype, a specialised motility that enables hyperflagellated bacteria to efficiently colonise surfaces and requires a combination of the chemotaxis, LPS synthesis, type III secretion and iron metabolism systems [26,27].

Biofilms have previously been studied with transcriptomic, proteomic and *in vivo *expression technology-based approaches for bacterial species, such as *E. coli *and *Pseudomonas *[28-30]. A large number of genes or proteins that are differentially regulated during biofilm development have been identified. Few studies have focused on the global response of *Salmonella *under environmental conditions relevant to food processing, where bacteria may encounter hydrodynamic stress and nutrient limitation [31]. Two studies have used a proteomic approach to identify *S*. Enteritidis proteins that are differentially regulated during biofilm growth in response to disinfectant and to different fluid flow rates [9,32]. However, the physiological and regulatory processes involved in the growth and persistence of *Salmonella *biofilms remain unclear. We have used a combination of physiological, transcriptomic and proteomic approaches to address this problem in *S*. Typhimurium.

## Results

### Growth of *Salmonella *biofilms

The biofilm phenotype of *Salmonella *isolates is known to vary significantly depending on the strain, nutrient source, temperature and other factors [23,33-35]. Because *Salmonella *can encounter hydrodynamic environments at several stages in food processing [32], we assessed the biofilm forming capacity of *S*. Typhimurium SL1344 on glass in a flowing system. We found that SL1344 produced substantially thicker biofilms in Colonising Factor Antigen (CFA) medium at 25°C, when compared to growth at 37°C, or growth in rich nutrient media (Luria Broth, LB), Brain Heart Infusion or M9 minimal media supplemented with glucose, sucrose or glycerol (data not shown). CFA medium has been previously shown to promote biofilm growth of *S*. Typhimurium [36].

SL1344 biofilms were then grown in a modified batch system for 72 h (Additional file 1) using silicone rubber tubing as a substratum for growth, as this surface permitted the isolation of sufficient quantities of mature biofilm and planktonic cells for proteomic and transcriptomic analyses (see Methods). The tubing was positioned vertically to avoid the isolation of bacterial aggregates following sedimentation. The pH of the medium effluent showed no detectable change (pH 7.0 ± 0.2) throughout the experiment. Influent samples were collected aseptically throughout the experiment showing that the number of cells increased from 1 × 10^6 ^CFU ml^-1 ^(T = 0) to 5 × 10^8 ^CFA ml^-1 ^(T = 72 h). No pellicle formation was observed in the influent flask during the 72 h experiment. To determine if the cells within a mature 72 h biofilm were metabolically active, an analogous biofilm grown in a glass flow cell was stained with Live/Dead BacLight. Images captured at ten random fields of view along the glass surface confirmed that more than 95% of the bacterial cells were alive after 72 h of growth (data not shown). Taken together, these experiments established that biofilms of SL1344 continued to accumulate biomass on glass for at least 72 h, and that the majority of these cells were viable.

### The global gene expression profile of *Salmonella *biofilm

The transcriptome of mature *S*. Typhimurium biofilm (72 h) was compared to its planktonic counterpart (72 h) in three independent biological experiments. Capillary gel electrophoresis was used to confirm that the RNA obtained from the 72 h biofilm and planktonic samples was of good quality prior to labelling (data not shown). The transcriptomic data from the three biological replicates were statistically filtered and data are only presented for genes that showed significant changes in every replicate.

**Figure 1:**
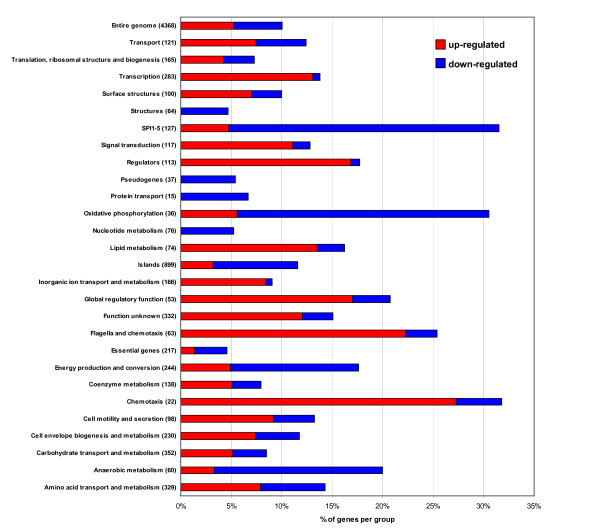
**Whole genome expression profiling of *S*. Typhimurium SL1344 flowing biofilms compared to planktonic cells when grown in CFA at 25°C for 72 h**. Expression changes of genes belonging to functional groups and pathogenicity islands (numbers in parenthesis refer to the genes assigned to each functional group from the genome of *S*. Typhimurium LT2). The bars show the percentage of genes belonging to each group that were altered for expression > 2-fold between planktonic and flowing biofilm cells. The blue bars indicate the proportion of genes that are down-regulated and the red bars represent the proportion of up-regulated genes for each group during biofilm growth (P < 0.01).

**Figure 2:**
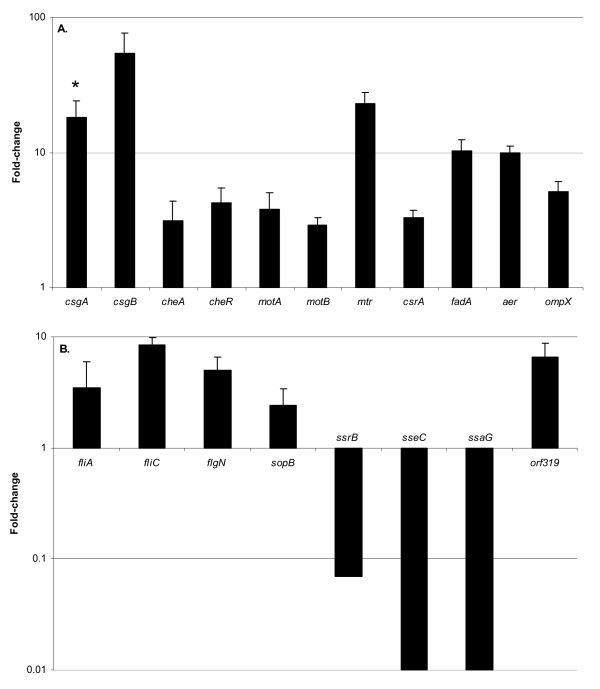
**Biofilm-regulated *S*. Typhimurium gene expression**. **(A) **A subset of genes up-regulated in the biofilm that encode cell surface structures, motility, global regulation, and oxygen diffusion. Representative examples of the functional categories (Figure 1) are shown. **(B) **Type III secretion genes. Transcriptomic data from the flowing biofilm system (72 h) was normalised to the planktonic samples and values are shown as fold change on a logarithmic scale (e.g. value of 10 on the Y-axis corresponds to 10-fold up-regulated). All genes were significant at P < 0.01 (* denotes that the *csgA *gene was significant at P < 0.05).

The transcriptomic data showed that a total of 560 genes were differentially expressed (P < 0.01) in biofilms compared with 72 h planktonic cultures, and 433 of these genes (10% of the *S*. Typhimurium genome) displayed more than a 2-fold change in expression. These included 229 genes that were up-regulated in biofilm (Additional file 2) and 204 down-regulated genes (Additional file 3). We noted that almost half of these genes were of hypothetical or unknown function. All differentially expressed genes were catalogued according to functional categories and were predominantly involved in cell motility, amino acid metabolism, stress response, outer membrane function and virulence (Figure 1). Figure 2A shows a subset of up-regulated genes that correspond to cellular processes previously implicated in biofilm growth (e.g. cell surface structures, motility, global regulation and oxygen availability) and Figure 2B shows a subset of genes specific to Type III secretion. The complete transcriptomic data sets are presented in Additional files 2 and 3, and the key findings are discussed below.

In *E. coli*, cell surface structures such as fimbriae have been shown to be required for the initial colonisation of abiotic and biotic surfaces and the establishment of well-established biofilms [37-40]. In mature biofilms of *S*. Typhimurium, several genes required for bacterial attachment and motility were up-regulated, including *csgB *and *csgA *that encode the curlin fimbrial subunits (Figure 2A). The extracellular matrix of *S*. Typhimurium biofilms is composed of curli, along with cellulose, colanic acid and other polymers, depending on nutrient availability [24]. Under the conditions used in this study, biofilm cells showed increased expression of one gene, *bcsE*, required for cellulose biosynthesis. Genes involved in flagellar biosynthesis, assembly and regulation were up-regulated by up to 7-fold (Figure 2B). Genes required for motility and chemotaxis were also up-regulated in *S*. Typhimurium biofilms, including *motAB, cheA*, *cheY*, *cheR *(Figure 2A) and *tsr*. Other cell-surface associated genes that were highly expressed in the biofilm included those encoding the major outer membrane protein OmpX (Figure 2A), a regulator of LPS O-chain length WzzB, the OM lipoprotein Blc, the membrane bound lipid phosphotase PgpB and 13 genes of hypothetical cell envelope function.

Gradients in oxygen concentration have been experimentally demonstrated within biofilm clusters [41]. Consistent with this, several genes that sense or respond to oxygen availability were up-regulated in flowing biofilm, including *aer *(Figure 2A) and *fnr *[42]. *cyo *genes encode the cytochrome o ubiquinol oxidase subunits of the aerobic respiratory chain, and are regulated by the master flagellar regulator [43]. The *cyo *genes were more highly expressed in the biofilm suggesting that the environment of the biofilm was aerobic. Furthermore, several genes known to be repressed by aerobiosis were down-regulated in the biofilm, including genes of the fumarate (*fum*, *frd*), hydrogenase 2 (*hyb*), cytochrome o (*cyd*) and dicarboxylate (*dcu*) operons (Additional file 3). While biofilms are reported to be spatially heterogeneous in terms of oxygen levels, our results are consistent with some penetration of oxygen into cell clusters in a flowing environment, as suggested by Xu *et al*., [44]. This may reflect the high oxygen permeability of the silicone surface used for growing our biofilms [45].

The precise growth phase of bacteria within the interior of a biofilm cell cluster has not been established, although several groups have reported slow growth rates, presumably due to nutrient limitation [16,46]. Specific operons involved in translation (i.e. *rps, rpl, rpm*) were down-regulated in biofilms of *S*. Typhimurium when compared to planktonic cells, suggesting that slower growth occurred in the attached population (Additional file 3). Transcriptomic comparisons with planktonic cells that were isolated at earlier time points of the experiment (i.e. 6 and 24 h), indicated that mature biofilm cells were most similar to a stationary phase planktonic population (data not shown). This observation is consistent with findings from studies in *E. coli *where biofilms cells have similarities to bacteria in stationary phase [37,40].

Global gene regulators respond to environmental conditions, including nutrient limitation, oxygen availability and osmotic stress, and control a wide range of adaptive physiological and regulatory circuits. Several genes that encode global gene regulators were up-regulated in flowing biofilms of *S*. Typhimurium, including *csrA *(Figure 2A) and *ihfA*, confirming previous studies in well-established biofilms of *Salmonella *or *E. coli *[47,48]. Other regulatory genes that respond to starvation conditions, including *rpoH*, *cbpA *and *phoH*, were up-regulated during biofilm growth (Additional file 2). RpoS (σ^38^), the sigma factor that activates genes under growth arresting conditions, was highly expressed in both biofilm and planktonic populations. We observed that more than 25% of the *S*. Typhimurium RpoS regulon [49] was up-regulated in biofilm cells (Additional file 2).

Following three days of growth, biofilms of *S*. Typhimurium showed up-regulation of genes that respond to oxidative stress (*lexA, msrA, soda, sodC, gloA*), heat shock (*clpA, clpX*), DNA replication and repair (*recA, mug, phrB*), cell envelope stress (*pspB*) and a putative stress-related gene (*yicC*) when compared to planktonic cells. A possible role for MsrA, RecA, PspB and the cytoplasmic Clp protease in biofilm development of Gram-negative species has been previously reported [37,50]. At least 19 genes that were up-regulated during biofilm growth of *S*. Typhimurium have a role in the osmotic stress and acid tolerance responses of *E. coli *[51], including *treF*, *talA*, *poxB*, *osmCY*, *himA*, *dps *and *aceB *(Additional file 2).

Amino acid synthesis is energetically expensive for the cell but essential for protein production, nitrogen transfer and osmotic protection [52]. We identified genes involved in alanine (*dad*) and glutamine/glutamate (*gln*, *gltL*, *astE*, *nadE*, STM1795) metabolism and transport that were more highly expressed in the biofilm (Additional file 2). We were intrigued to discover that the biosynthetic genes of the *trp *operon were over-expressed in well-established biofilms of *S*. Typhimurium (Table 1). These genes are required from the initial steps of tryptophan synthesis (i.e. from chorismate to indole) to the transfer of indole to tryptophan [53]. Additionally, there was a strong biofilm-dependent induction of *mtr*, a tryptophan-specific transporter (Figure 2A). Recent studies reported the induction of tryptophan biosynthesis genes during early biofilm formation in *E. coli*, followed by repression of this operon at later time points [38,40].

**Table 1:** Expression levels of *S*. Typhimurium tryptophan biosynthetic genes during biofilm growth under flowing conditions.

Gene name	Description	Identifier	Fold-induction	*P*-value^a^
*trpD*	Anthranilate synthase, component II	STM1724	5	3E-03
*trpC*	Tryptophan biosynthesis protein TRPCF, bifunctional	STM1725	11	4E-04
*trpB*	Tryptophan synthase beta protein	STM1726	8	6E-05
*trpA*	Tryptophan synthase alpha chain	STM1727	7	7E-05
*trpS2*	Putative tryptophanyl-tRNA synthetase	STM4508	2	6E-04
*trpR*	Transcriptional regulator for tryptophan operon and *aroH*	STM4583	3	3E-04

a. Genes which passed the statistical filtering (P < 0.01). Expression values are expressed as fold-induction comparing expression in biofilm cells to planktonic cells.

Expression profiles indicated that virulence genes located within *Salmonella *pathogenicity island 1 (SPI1) and the serotype-specific *S*. Typhimurium plasmid (pSLT) were not differentially expressed during biofilm growth. However, more than 30 genes belonging to the *ssr*, *ssa *and *sse *operons of SPI2, were down-regulated by up to 100-fold in the biofilm (Figure 2B; Additional file 3); SPI2 encodes a type III secretion system that is required for intracellular survival in host phagocytes [54]. This prompted us to monitor the level of SPI2 expression observed in our CFA media-based experiments at 25°C compared with growth in LB at a higher temperature (data not shown). Surprisingly, our transcriptomic data showed that SPI2 was expressed at a significantly higher level in planktonic CFA cultures grown at 25°C than at mid-log phase in LB at 37°C. This suggests that the CFA media contains a factor that induces SPI2 expression, even at this low temperature (data not shown). The only SPI2 gene observed to be up-regulated in biofilm was *orf319*, which encodes a putative transmembrane protein of unknown function and does not contribute to *S*. Typhimurium virulence (Figure 2B) [55]. In addition, *sopB*, a SPI5-encoded phosphatase required for invasion of epithelial cells [56], was up-regulated in *Salmonella *biofilm.

### Surface-associated growth of *S*. Typhimurium leads to changes in protein expression

**Figure 3:**
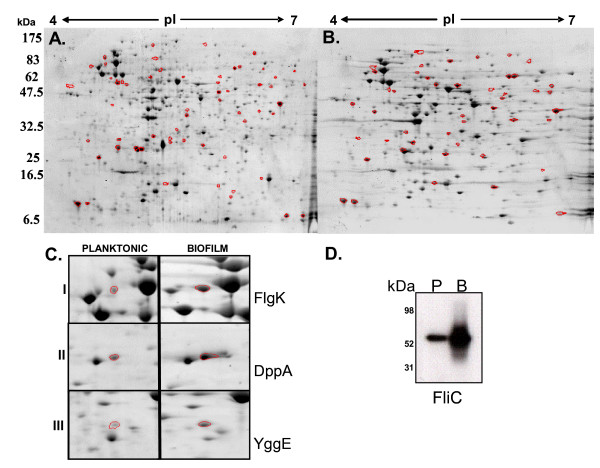
**Biofilm-regulated protein expression**. SYPRO^® ^Ruby stained 2-D gels of total protein extracts from flowing biofilm **(A) **and planktonic **(B) **cells of *S*. Typhimurium grown in CFA medium at 25°C for 72 h. Spots circled in red were excised from the gels and identified by mass spectrometry and peptide mass fingerprinting. **(C) **Magnification of 2-D gels comparing expression of FlgK **(I)**, DppA **(II)**, and YggE **(III)**, which were all more highly expressed in the biofilm cells than in planktonic cells. **(D) **Western immunoblot of total protein extracts of mature *S*. Typhimurium biofilm (B) and planktonic (P) cells both grown in CFA medium for 72 h. Protein extracts were separated on a denaturing SDS-PAGE gel and probed with anti-FliC monoclonal antibody. Densitometric analysis showed that FliC was 3.4 fold induced in the biofilm compared with planktonic cells.

To expand our analysis from the transcriptomic to the proteomic level, and to identify biofilm-regulated proteins, total protein extracts from biofilm and planktonic populations of *S*. Typhimurium were compared using 2-D PAGE. Samples from the same flowing biofilm system were used for the proteomic and the transcriptomic experiments. A representative example of the biofilm and planktonic proteome with over 600 detected proteins spots per gel is shown in Figure 3AB. Direct comparison of protein spots showed that the levels of at least 250 proteins remained similar in both populations. However, the expression of 124 proteins was altered (> 2-fold), with the expression of 59 proteins increasing and 65 proteins decreasing during surface-associated growth compared to planktonic culture. At least 175 proteins were only detected in the biofilm samples, and not in planktonic culture.

### Identification of differentially regulated and unique proteins

Fifty protein spots that showed differential expression between biofilm and planktonic gels, as well as spots detected as unique to either mode of growth (Figure 3AB), were selected and analysed by MALDI-TOF mass spectrometry (MS). These spots were specifically chosen as they were abundant and clearly separated from other spots, to facilitate unambiguously identification by MS. Forty-four proteins were successfully identified, and we discovered that the majority of proteins that were unique or highly expressed in the biofilm corresponded to the same functional groups identified by transcriptomic analyses. These included proteins involved in cell motility, amino acid and carbohydrate metabolism, as well as proteins of unassigned function (Additional file 4). In fact, 45% of the 24 up-regulated proteins corresponded to genes identified as differentially expressed at the transcriptional level (Table 2). A common theme emerged relating to cell motility, with the up-regulation of three external flagellar proteins FlgK (Figure 3C), FlgL and FliD (also known as HAP1, HAP3, and HAP2) that are involved in the later stages of flagellar assembly. FlgK and FlgL were up-regulated in the biofilm compared to planktonic growth and FliD was only detected in the biofilm samples. Our transcriptomic analysis showed an increase in the expression of FliC in the biofilm, but this protein was not identified in the 2-D analysis. To confirm the biofilm-dependent regulation of proteins involved in late flagellar assembly, we performed a Western blot. As shown in Figure 3D, FliC protein was 3.4-fold more abundant in biofilm cells when compared with a planktonic culture.

**Table 2:** Biofilm-regulated proteins that show similar trends in proteomic and transcriptomic analysis.

Protein^a^	Function	Average fold expression in the biofilm compared to planktonic (n = 2)
DppA	ABC superfamily dipeptide transport protein	**+ 10.7**

TreA	Periplasmic trehalase	**Unique to biofilm**

GalE	UDP-galactose-4-epimerase	**+ 2.9**

YggE	Putative periplasmic immunogenic protein	**+ 3.7**

YciF	Putative cytoplasmic protein	**Unique to biofilm**

YajQ	Putative cytoplasmic protein	**Unique to biofilm**

FlgL	Flagella hook-filament junction protein (HAP3)	**+ 44.0**

IadA	Putative isoaspartyl dipeptidase	**Unique to planktonic**

FliD	Flagellar filament cap protein (HAP2)	**Unique to biofilm**

FlgK	Flagellar hook-filament junction protein (HAP1)	**+ 3.3**

AnsB	Periplasmic L-asparaginase II protein	**+ 2.9**

ArgT	ABC superfamily; lysine/arginine/orthinine tranport protein	**+ 4.9**

a. Proteins identified by MALDI-TOF mass spectrometry and peptide mass fingerprinting (Mascot). Proteins up-regulated in the biofilm that show the same pattern of expression at the transcriptional level (Additional files 2 and 3).

Two periplasmic transport proteins were up-regulated in biofilm, namely DppA (10-fold; Figure 3C), a dipeptide-binding protein and ArgT (5-fold), which transports arginine, lysine and orthinine. Proteomic analysis of *S*. Typhimurium biofilm cells also showed differential expression of AnsB, TreA and GalE which are involved in asparagine metabolism, trehalose degradation and the conversion of UDP-galactose and UDP-glucose (Additional file 4). The remaining proteins that were unique or more highly expressed during biofilm growth were of unknown function (Additional file 4). The putative periplasmic protein YggE, which is highly conserved in Enterobacteriaceae and has been shown to possess immunogenic properties in *Edwardsiella ictaluri *[57], was also more abundant (3.7-fold) during biofilm growth (Figure 3C).

### Targeted deletion of biofilm-regulated genes

To investigate the function of genes shown to be differentially expressed in *S*. Typhimurium biofilm, eight targeted gene deletions were constructed (Additional file 5AB). Genes that showed a biofilm-specific pattern of expression were selected for chromosomal mutation, including *mtr*, *yhfG*, *ybaY *and genes of the *trp *operon (Additional file 6). Disruption of *trpE*, which encodes anthranilate synthase component I, was chosen because four genes of the tryptophan operon were highly up-regulated during biofilm growth and the product of *trpE *catalyses the first reaction of the tryptophan pathway with TrpD (Table 1). Moreover, TrpE is the most important enzyme of this pathway from a regulatory point of view, as it is subject to feedback inhibition by tryptophan [53]. The other four genes were selected on the basis that they were they most highly expressed (*ygaT*) or repressed (STM0341, STM2779) in the biofilm, or because they were previously implicated in biofilms of *E. coli *(*pspABCDEF*) [37]. To test whether the chromosomal deletions altered the fitness of the strains, the growth patterns of each mutant were analysed over 24 h in CFA and LB medium. No significant differences were observed in the planktonic growth rate of each mutant compared to the SL1344 parental strain (Additional file 7).

**Figure 4:**
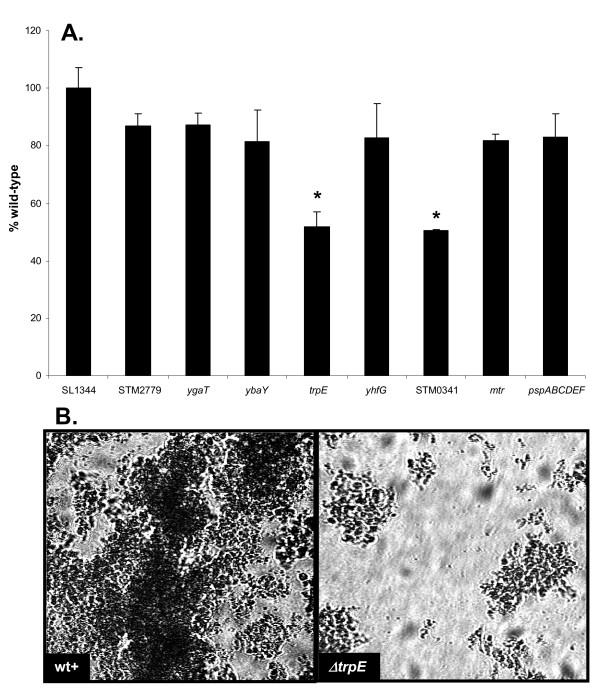
**(A) Static biofilm formation of eight targeted gene deletion mutants, compared to attachment of WT SL1344**. Following incubation at 25°C for 24 h in CFA medium, the level of biofilm formation is expressed as a percentage of WT SL1344 which had an A_590 nm _of 0.56 ± 0.04 in this experiment. The Δ*trpE *(JH3185) (* P = 0.0001) and ΔSTM0341 (JH3187) (* P = 0.001) mutants showed significantly less attachment to polystyrene than WT SL1344. The mean absorbance values from four wells are shown as a percentage of WT SL1344 and the error bars represent the SD between four technical replicates (n = 3). **(B) **The attachment of the Δ*trpE *(black diamonds) mutant (JH3185) to the bottom surface of a glass flow cell compared with *S*. Typhimurium SL1344 (back circles). Bacteria were cultured at 25°C in CFA medium in a glass flow cell. Error bars represent the standard deviation between 6 images captured along the length of the flow cell over 24 h (n = 3).

To determine whether the deletion of these genes altered biofilm formation, each strain was tested for its ability to attach to polystyrene in a static biofilm assay. As shown in Figure 4A, the Δ*trpE *mutant formed significantly less biofilm (P = 0.0001) than the parental strain after 24 h, showing that anthranilate synthase is required for biofilm formation. Similarly, biofilm growth of ΔSTM0341 was significantly reduced (P = 0.001). None of the other mutations affected growth of biofilms.

To determine whether the Δ*trpE *mutant also had a biofilm defect on a hydrophilic glass surface, biofilm formation was compared to WT SL1344 in a once-through glass flow cell system. The attachment of Δ*trpE *was monitored by light microscopy for 24 h and these results confirmed that the mutant formed reduced levels of biofilm (P < 0.01; Figure 4B; n = 3). Although Δ*trpE *cells did transiently attach and form small microcolonies on glass, they failed to grow and to form thick cell clusters on the flow cell surface, resulting in 3-fold less of the surface being covered with biofilm compared with the WT strain (Figure 4B). The biofilm forming capacity of the ΔSTM0341 mutant was also reduced on glass when compared to WT SL1344 in one independent experiment (data not shown).

### Inactivation of *ssrA *and *rpoS *alters biofilm formation in *S*. Typhimurium

**Figure 5:**
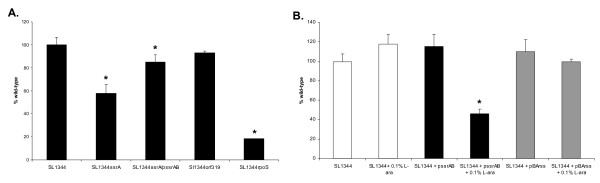
**Static biofilm formations**. **(A) **Static biofilm formation of SPI2 and *rpoS *deletion mutants. Following incubation at 25°C for 48 h in CFA medium, the level of biofilm formation is expressed as a percentage of WT SL1344 which had an A_590 nm _of 0.15 ± 0.02 in this experiment. The Δ*ssrA *(JH3180) and Δ*rpoS *(JH3142) mutants showed significantly less attachment to polystrene than WT SL1344 after 24 h (data not shown) and 48 h of growth (* P = 0.001-0.02). Complementation of Δ*ssrA *with a low copy plasmid encoding *ssrAB *(JH3181) restored the ability of this mutant to form WT biofilm after 48 h (* P = 0.002). The mean absorbance values from four wells are shown as a percentage of WT SL1344 and the error bars represent the SD between 4 technical replicates (n = 2). **(B) **Static biofilm formation of SL1344 over-expressing SsrAB (p1437-1) in CFA medium (+ 0.1% L-arabinose where indicated) for 24 h at 25°C. The level of biofilm formation is expressed as a percentage of WT SL1344, which had an A_590 nm _of 0.14 ± 0.01 in this experiment. The induction of SsrAB expression by an arabinose-inducible promoter significantly inhibited attachment (* P = 0.000007) when compared to WT SL1344. No significant difference in attachment was observed in the control strain over-expressing SsrAB in the reverse orientation (p1437-6). The mean absorbance values from six wells are shown and the error bars represent the SD between 4 technical replicates.

Our transcriptomic analysis revealed that genes of the SPI2 type 3 secretion system were differentially expressed (i.e. down-regulated up to 100-fold) during biofilm growth. SPI2 repression is activated by the two-component regulatory systems SsrAB and PhoP/Q and in response to high levels of phosphate and Mg^2+^, however this repression during biofilm growth has not been previously reported [58-61]. To determine the effect of SPI2 gene expression on *Salmonella *biofilm formation, the ability of a Δ*ssrA *deletion mutant to attach to polystyrene was compared to WT SL1344. The same Δ*ssrA *strain has been used to identify the SsrA regulon of *S*. Typhimurium [62]. A Δ*orf319 *deletion mutant was also tested as this was the only SPI2-associated gene that was up-regulated in the biofilm. No significant differences were observed in the planktonic growth rates of Δ*ssrA *or Δ*orf319 *compared to the SL1344 parental strain (Additional file 7). Deletion of *ssrA *caused more than a 40% decrease in attachment after 24 h and 48 h of growth, and this was restored to WT levels by complementation with a low copy plasmid encoding *ssrAB *(Figure 5A). The Δ*orf319 *mutant did not show a significant defect in biofilm formation. To further examine the effect of SPI2 expression and its impact on biofilm formation, we over-expressed *ssrAB *from an arabinose-inducible plasmid (Figure 5B). These results showed that increased SPI2 expression significantly reduced biofilm formation in *S*. Typhimurium.

Transcriptomic comparison of biofilm and planktonic cells showed that *rpoS *was highly expressed in both populations, and that several RpoS-activated genes showed biofilm-specific patterns of expression. To determine the impact of RpoS expression on biofilm growth, we tested the fitness of a Δ*rpoS *mutant compared to WT SL1344 and its ability to colonise polystyrene (Additional file 7, Figure 5A). We found that inactivation of *rpoS *significantly reduced the ability of *S*. Typhimurium to form a biofilm (P < 0.0001).

### Phenotypic characteristics of *S*. Typhimurium deletion mutants

The biofilm-forming capacity of *S*. Typhimurium strains has been linked to the expression of curli and cellulose production [21]. Likewise, the ability of *E. coli *to colonise and grow on surfaces has been shown to require flagella and RpoS [46,63]. Because of the importance of these cellular functions for biofilm formation, we examined whether the mutant strains showed alterations in swimming motility, RpoS activity or the ability to bind calcofluor and congo red (CR) dye, compared with the WT strain (Table 3). All deletion mutants showed the same pattern of flagellar-mediated motility as WT SL1344, indicating that the reduced biofilm formation observed in Δ*trpE, Δ*STM0341 or Δ*ssrA *did not reflect a motility defect. When RpoS activity was examined indirectly by assessment of KatE-encoded catalase production, all of the mutants showed the same RpoS-positive phenotype as the WT SL1344 strain.

**Table 3:** Phenotypic characteristics of deletion mutants and WT strain tested for motility, RpoS activity, calcofluor binding and changes in extracellular matrix.

Strain	Genotype	Biofilm Capacity^a^	Motility	RpoS activity^b^	Calcofluor binding^c^			EPS production		
					25°C	37°C	25°C^d^	Morphology	37°C^d^	Morphology
SL1344	wild-type	+	+	+	L	L	DR	R	DR	R
JH3185	Δ*trpE*	---	+	+	L	L	DR	S	R	R/S
JH3187	ΔSTM0341	---	+	+	L	L	DR	R	DR	R
JH3180	Δ*ssrA*	---	+	+	L	H	DR	R	DR	R
JH3181	Δ*ssrA/pssrAB*	+	+	+	L	H	DR	S	DR	S
JH3179	ΔORF319	+	+	+	L	H	DR	R	DR	R
JH3182	ΔSTM2779	+	+	+	H	L	DR	S	DR	R
JH3183	Δ*ygaT*	+	+	+	H	L	DR	R	DR	R
JH3184	Δ*ybaY*	+	+	+	H	L	DR	R	DR	R
JH3186	Δ*yhfG*	+	+	+	H	L	DR	R	DR	R
JH3188	Δ*mtr*	+	+	+	H	L	DR	R	DR	R
JH3189	Δ*pspABCDEF*	+	+	+	H	L	DR	R	DR	R
JH3142	Δ*rpoS*	---	+	---	---	---	W	R	W	S

a. Biofilm formation is based on attachment under static conditions after 24 and 48 h of growth in CFA medium at 25°C. Symbols: (+) = WT biofilm growth; (-) = severely reduced attachment.

b. RpoS activity as defined by catalase activity in the semi-quantitative assay as described by Zambrano *et al*., [106].

c. Calcofluor binding was assessed following growth of SL1344 and mutant strains at 25 and 37°C on CFA agar supplemented with fluorescent brightener 28. Fluorescence levels are reported as low (L) or high (H) as compared to a cellulose-deficient *rpoS *mutant (-).

d. Colony colour was reported as dark red (DR), red (R), or white (W) with dark red indicating WT EPS production. Colony morphology was assessed as rough (R) or smooth (S) following overnight growth on CFA agar supplemented with CR dye at 25 and 37°C. Colony morphology of WT SL1344 and mutant strains were compared to strain positive (*S*. Typhimurium LT2) and negative (Δ*rpoS*) for the rdar morphotype.

Calcofluor has previously been used to detect cellulose-producing strains of *S*. Typhimurium as this dye binds polysaccharides with 1,4β-glucopyranosyl units and fluoresces under UV light [24] To assess cellulose production, we grew each strain on CFA Calcofluor agar at 25 and 37°C and compared them to WT SL1344 and the cellulose-deficient *rpoS *mutant [64]. Six of the strains that were positive for biofilm formation on CFA (i.e. ΔSTM2779, Δ*ygaT*, Δ*ybaY*, Δ*yhfG*, Δ*mtr *and *ΔpspABCDEF*) fluoresced brightly at 25°C. In contrast, three of the mutants (ΔSTM0341, Δ*trpE*, and Δ*ssrA*) that exhibited impaired levels of biofilm formation showed lower levels of calcofluor binding at 25°C, suggesting reduced levels of cellulose production (Table 3). The Δorf319 mutant also showed lower levels of binding to calcofluor but was not impaired in biofilm formation. Experiments to monitor cellulose production at 37°C showed that Δ*ssrA, ΔssrA/pssrAB *and *Δ*orf319 fluoresced more brightly than any other strain, suggesting a temperature-dependent production of cellulose in these SPI2 deletion strains.

Co-expression of genes specific to EPS, curli and cellulose production results in the rdar colony morphotype in *S*. Typhimurium [64]. This morphotype is characterised by the binding of CR dye and the formation of red, dry and rugose spreading colonies. In strains not expressing curli and cellulose, a conventional smooth white colony is observed. We analysed the appearance of each strain on CFA-CR agar at 25°and 37°C compared to strains positive (*S*. Typhimurium LT2) and negative (Δ*rpoS*) for the rdar morphotype [64,65]. Incubation at 25°C resulted in SL1344 and most of the mutants producing the rdar morphotype (Table 3). Three strains (Δ*trpE*, ΔSTM2779, Δ*ssrA/pssrAB*) showed altered morphology (i.e. smooth dark red colonies) at 25°C. However, the smooth phenotype disappeared when Δ*trpE *and ΔSTM2779 were grown on CR at 37°C. These results indicate that chromosomal deletion of *trpE*, STM2779 (and *rpoS*) conferred temperature-dependent changes in EPS production, which were only apparent at 25°C. We noted that the Δ*trpE *and Δ*rpoS *mutants showed a reduced ability to colonise polystyrene at 25°C, the same temperature at which a smooth or white colony phenotype was produced.

Previous work showed that the *tnaA *gene, encoding for tryptophanase, was required for *E. coli *biofilm formation on abiotic surfaces and human pneumocytes, and speculated that TnaA may modulate pH changes during attachment [66]. Since the *tnaA *gene is not present in *S*. Typhimurium, we investigated whether the Δ*trpE *and ΔSTM0341 mutants, which showed reduced colonisation of abiotic surfaces and cellulose production, also showed altered levels of adherence or invasion of epithelial cells. We found that the ΔSTM0341 mutant was 9-times less adherent and 4-times less invasive than its parental strain (P < 0.01; data not shown), however *Salmonella *derivatives lacking the *trpE *gene did not show a significant difference in adherence and invasion compared to WT SL1344.

### A role for aromatic amino acids in *Salmonella *biofilm growth

**Figure 6:**
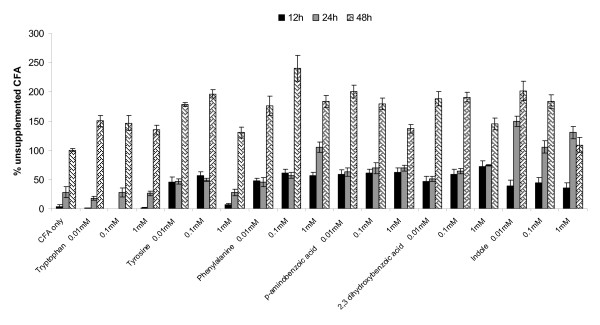
**The effect of aromatic amino acids on static biofilm formation of wild-type *S*. Typhimurium**. CFA medium was supplemented with increasing concentrations of aromatic amino acids. The level of biofilm formation after 12, 24 and 48 h of growth at 25°C is expressed as a percentage of the biofilm formed after unsupplemented growth in CFA at 48 h, which had an A_590 nm _of 0.15 ± 0.02 in this experiment. The mean absorbance values from four wells are shown and the error bars represent the SD between 4 technical replicates.

Our data showed that inactivation of the tryptophan biosynthetic pathway altered the biofilm-forming capacity and EPS production by *S*. Typhimurium. To investigate the impact of amino acid availability on surface attachment, SL1344 was grown in CFA broth supplemented with different concentrations of aromatic and non-aromatic amino acids. Biofilm formation was determined after 12, 24 and 48 h of growth and compared to non-supplemented medium. Casamino acids, the main constituent of CFA medium, have been used in nutritional studies to determine bacterial growth requirements for peptides and amino acids [67]. Supplementation with non-aromatic amino acids did not alter the attachment of *S*. Typhimurium to polystyrene (data not shown), whilst a significant increase in biofilm formation occurred in the presence of aromatic amino acids (Figure 6). This positive effect was noted even after 12 h of incubation, where the addition of all aromatic compounds, apart from tryptophan, significantly increased the number of adherent cells from 10 to 15-fold when compared with non-supplemented medium (P < 0.005). Similar positive effects on biofilm formation were observed after 24 h, with the greatest increase noted in wells containing indole (P < 0.001). Interestingly, supplementation with tryptophan had significant effects on surface associated growth at the later stages of biofilm development (i.e. 48 h). The addition of all aromatic amino acids significantly enhanced biofilm formation after 48 h of incubation, compared with non-supplemented CFA (P < 0.005).

### Tryptophan and indole rescue the biofilm defect of the *ΔtrpE *mutant

**Figure 7:**
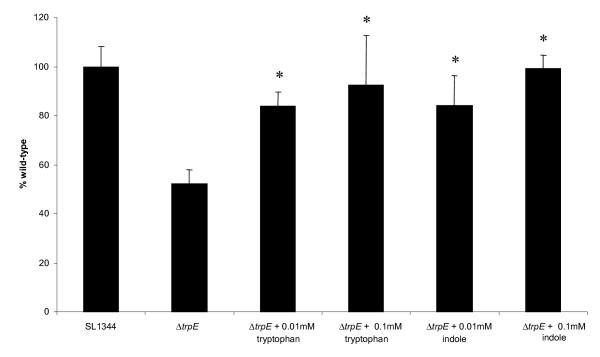
**Tryptophan biosynthesis is required for biofilm formation**. The effect of the *trpE *mutation and addition of tryptophan (0.01 mM, 0.1 mM) and indole (0.01 mM, 0.1 mM) on static biofilm growth of Δ*trpE *(JH3185) was determined after 24 h of growth in CFA broth at 25°C. The addition of tryptophan and indole significantly (*P = 0.02-0.00003) increased biofilm formation when compared to Δ*trpE *grown in CFA alone. The mean absorbance values from four wells are shown and the error bars represent the SD between 4 technical replicates.

To determine whether the phenotypes associated with the *ΔtrpE *mutation were the direct result of cells being unable to synthesise or take up sufficient tryptophan or indole, the effect of these aromatic compounds on biofilm formation by Δ*trpE *was investigated (Figure 7). Again, biofilm formation by the Δ*trpE *strain was significantly reduced, when compared to SL1344 (P = 0.0001). The addition of tryptophan to the Δ*trpE *mutant significantly increased biofilm formation, compared to growth in CFA alone (P = 0.01). In fact, the addition of 0.1 mM tryptophan completely restored the ability of the Δ*trpE *strain to form a WT level of biofilm. Biofilm development by the Δ*trpE *strain was significantly higher after supplementation with indole, compared to growth in CFA alone (P = 0.008). The highest concentration of indole (i.e. 0.1 mM) completely restored the ability of the Δ*trpE *strain to form a biofilm on polystyrene. These findings are strikingly different to the situation for *E. coli *where exogenous indole reduces biofilm formation [68].

## Discussion

It is clear that the ability of *S*. Typhimurium to grow as a biofilm on foods and processing surfaces represents an important survival strategy [2,3,13,69,70]. We found that both nutrients and temperature had a profound effect on the attachment of *S*. Typhimurium SL1344. In this study biofilms were grown at 25°C, resulting in extensive colonisation of abiotic surfaces. Our finding that 95% of cells remain viable within established sessile communities of *S*. Typhimurium confirms that biofilms represent a potential reservoir for infection.

We used transcriptomic and proteomic approaches to analyze the changes that occur in *S*. Typhimurium in response to biofilm growth in the type of low nutrient, hydrodynamic environment that can be found during the processing of chickens [2,3,69]. Transcriptomic analysis revealed the differential expression (i.e. more than a 2-fold change) of about 10% of *S*. Typhimurium genes during biofilm growth. The 229 genes that were significantly up-regulated during biofilm growth included several genes previously implicated in biofilm development of *S*. Typhimurium, including curli, cellulose and genes required for motility [23,24,71], confirming the link between gene expression and gene function in our experiment. Although the value of whole genome studies for the comparison of heterogeneous biofilms has been a subject of debate, transcriptomic approaches have provided great insight into *E. coli *biofilm biology [72]. Prior to undertaking this study, the technical and biological reproducibility of a microarray-based approach for comparison of biofilm and planktonic *S*. Typhimurium cells was shown to be robust [73].

Previous transcriptomic studies in other bacterial species showed that biofilm growth results in differential regulation of between 1% to 14.5% of the bacterial genome. While genetic variation between the organisms studied thus far may account for a proportion of these differences, we believe that the wide disparities between the proportions of biofilm-regulated genes reflect different experimental approaches. For example, cells were harvested at different times from different growth media and dissimilar model systems were used to cultivate the biofilm cells. Such technical differences greatly impact upon gene expression, and therefore the identification of biofilm-regulated genes, complicating the comparison of transcriptomic data between studies [74]. A critique of the experimental approaches used to study biofilms has recently been presented [75].

Proteomic analysis identified 24 proteins that were up-regulated in the biofilm, and 45% of these corresponded to genes that were differentially expressed at the transcriptional level. Proteins required for late flagellar assembly were up-regulated in biofilms, when compared to planktonic cells. Proteomic profiling of *P. putida *identified the same pattern of up-regulation of flagellar genes within mature biofilms [76]. In *S*. Enteritidis, thirty-two proteins were identified that showed differential expression in biofilms when compared to planktonic cultures [32], including down-regulation of flagellar proteins and different patterns of expression of ArgT and Crr which are at variance with our study. The differing planktonic populations used for comparison may explain these conflicting reports [29]. It remains unclear whether flagella expression is limited to initial surface attachment or if it allows bacteria to move in and around the biofilm cell clusters and colonise new areas during the later stages of biofilm development [77].

Unlike the proteomic approaches, transcriptomic studies have given several consistent messages; four studies comparing transcriptomic profiles of biofilm and planktonic-grown *E. coli *reported that biofilm growth leads to the up-regulation of genes involved in cell surface structures, amino acid metabolism, stress responses and anaerobic respiration [37-40]. Our data suggest that mature biofilms of *S*. Typhimurium and *E. coli *involve similar physiological modifications, supporting the hypothesis that some adaptations required for biofilm growth and survival are conserved between bacterial species.

Our analysis showed that many of the RpoS (σ^38^)-activated genes were up-regulated, suggesting that this regulon is important for the survival of *Salmonella *cells within the complex biofilm environment. Similar observations were made in *E. coli *biofilms [39,46], and RpoS is known to regulate the genes involved in curli and cellulose production [64]. We used an RpoS-deficient mutant to confirm that RpoS plays a crucial role in mature biofilms of *S*. Typhimurium.

Previous transcriptomic analysis of *E. coli *cells showed that many biofilm-regulated genes were of unknown function [28]. We found the same to be true in mature *S*. Typhimurium biofilms, with more than half of the differentially expressed genes having only putative or unknown function. Interestingly, seven of these genes, including *ydcI*, *yebE *and *yceP*, were also up-regulated in *E. coli *biofilms and the deletion of *yceP *has been shown to impair biofilm formation [37,38,78].

To identify genes that were directly involved in biofilm growth, we mutated the five uncharacterised genes that were most highly up- and down-regulated. Our results suggest that STM0341, a putative inner membrane protein, is required for biofilm growth in *S*. Typhimurium SL1344. Little is known about the function of STM0341 except that it shows significant homology to putative transmembrane regulators in *S*. Typhi and 25% amino acid identity to ToxR of *Vibrio cholerae *(Colibase accessed Oct 2009). Interestingly, the ΔSTM0341 mutant was 9-times less adherent and 4-times less invasive in epithelial cells than its parental strain, raising the possibility that expression of STM0341 could be directly or indirectly involved in efficient penetration of the gastrointestinal epithelial cell lining.

Table 3 summarises the data from biofilm growth and further phenotypic analyses of the other uncharacterised mutant strains (i.e. STM2779, *ygaT*, *ybaY*, *yhfG*). Several of the mutations did not confer a detectable change in biofilm growth, or flagella, curli and cellulose production, although the WT genes were highly differentially regulated in the biofilm. Our observation that several individual genes were not required for biofilm formation by *S*. Typhimurium is consistent with biofilm formation involving multiple pathways [50] or overlapping functions, as has been observed for the oxidative stress response of *S*. Typhimurium [79,80].

The transcriptomic analysis revealed that genes involved in amino acid metabolism were up-regulated during biofilm growth, particularly tryptophan biosynthesis genes. The requirement for tryptophan was confirmed at the phenotypic level by showing that a *trpE *mutant formed significantly lower levels of biofilm. Domka and colleagues [40] reported that tryptophan biosynthesis only occurred during early *E. coli *biofilm formation (< 7 h) in LB medium and that repression of the *trp *operon was required during the later stages, suggesting that low intracellular levels of indole are required for biofilm development. This was confirmed by repression of the indole uptake gene (*mtr*) and up-regulation of indole efflux genes (*acrEF*) in *E. coli *[40]. Such observations were consistent with earlier work showing that the absence of the regulatory YceP protein increased biofilm formation in *E. coli *by repressing genes that control indole transport into the cell and that a *trpE *mutation showed increased biofilm formation [78]. We have reported several similarities between our data and biofilm-specific regulation in *E. coli*, but our findings suggest major differences in tryptophan metabolism between *Salmonella *and *E. coli *biofilms. We have shown that tryptophan biosynthesis plays a role at the late stages of biofilm development in *S*. Typhimurium, and that *mtr *and *yceP *are both up-regulated in the mature biofilm. The precise function of tryptophan and indole during biofilm formation of *E. coli *remains to be completely elucidated. It is clear that the tryptophanase (*tna*) operon that converts tryptophan to indole is required for biofilm formation in *E. coli *and other indole-producing bacteria [66,81].

Our data showed that exogenous tryptophan or indole restore the ability of the *S*. Typhimurium *trpE *mutant to form a biofilm. Moreover, both tryptophan and indole increased the biofilm-forming capacity of WT SL1344; in fact, low levels of indole increased attachment at all of the time points tested. We speculate that the highest concentration of indole (1 mM) used in this study is not biologically relevant, and imposes stress on the cell. While there are no other published reports on the effect of indole concentration upon biofilm formation in *S*. Typhimurium, lower levels of exogenous indole (312 to 625 μM) induced biofilm formation in *E. coli*, while higher levels of exogenous indole (1250 μM) caused indole toxicity and decreased bacteria growth [81]. Taken together, these results suggest that in the absence of tryptophan, indole activates genes or pathways that contribute to biofilm formation.

Several reports have shown that indole acts as a signalling molecule in *E. coli *to: 1) prepare cells for a nutrient-poor environment and increase catabolism of amino acids, 2) up-regulate genes that encode drug exporters (e.g. *acrDE*, *mdtAE*, *cusB*) to increase bacterial tolerance to toxic compounds, 3) increase bacterial adherence to surfaces, and 4) delay cell division [81-84]. All of these functions would be beneficial to cells within a well-established biofilm. Indole has recently been shown to act as an interspecies signal that controls biofilm formation by acting on oxygenases of bacteria that do not synthesise this molecule at temperatures below 30°C [85,86]. It is possible that we have observed a similar cell signalling phenomena in *Salmonella*. Several Gram-negative bacterial biofilms have been shown to secrete the amino acid valine that may play a role in signalling or function as a protective osmolyte [87]. Further functional analysis of biofilms grown in the presence of exogenous tryptophan (and indole) should provide insights into the role of these molecules in *S*. Typhimurium.

Microbial biofilms are inherently more resistant to host defences and antimicrobials than planktonic cells, but it is not known what proportion of this phenotype is due to bacterial factors such as the expression of virulence proteins, or external environmental influences such as the diffusion of nutrients or oxygen, or slow growth rate. In this study, we report that mature biofilm growth in *S*. Typhimurium leads to significant down-regulation (up to 100-fold) of certain virulence genes located in SPI2. We confirmed the link between SPI2 expression and biofilm growth by showing that the SPI2-deficient Δ*ssrA *strain formed significantly less biofilm than the parental strain, and that complementation with *ssrAB *effectively restored attachment. We discovered that over-expression of SPI2 led to a significant reduction in bacterial attachment by the WT strain. These data show that both increased and decreased expression of SPI2 interferes with biofilm formation. It is not clear how a TTSS impacts upon biofilm formation, but we note that it has been reported that an extracellular molecule (LPS) can interfere with the TTSS-mediated attachment of *Shigella flexneri *to mammalian cells [88]. By analogy, we speculate that aberrant expression of the SPI2 TTSS apparatus compromises the ability of *Salmonella *to form biofilm, perhaps by affecting the presentation of cell surface factors such as curli.

## Conclusions

We have shown that biofilm growth of *S*. Typhimurium involves processes that include amino acid metabolism, motility, and virulence. Three proteins, TrpE, STM0341 and SsrA, play a role in biofilm formation by *S*. Typhimurium. We have discovered that tryptophan metabolism is required for effective biofilm formation in our experimental system. The unexpected link between SPI2 expression and biofilm promises to be a fertile area for *Salmonella *research in the future. Further characterization of the mutations that led to a reduction in biofilm growth is ongoing and we are examining temporal expression within the biofilm using a GFP reporter system. We are now comparing *S*. Typhimurium biofilms grown in dissimilar model systems to identify the core genes that are required for survival of this pathogen on different surfaces.

## Methods

### Bacterial strains, plasmids and growth conditions

Bacterial strains and plasmids used in this study are described in Table 4. Liquid growth media were Colonising Factor Antigen (CFA) medium comprised of (w/v) casamino acids (Difco) 1.0%, yeast extract (Difco) 0.15%, MgSO_4 _0.005% and MnCl_2 _0.0005% [89], Brain-Heart Infusion (BHI; Oxoid), Luria Broth (LB) or M9 minimal medium supplemented with glucose, sucrose or glycerol at concentrations of 0.2, 2.0 and 20% (v/v), according to Sambrook [90]. Bacto agar (Difco; 1.5% w/v) was used to make plates from these media, as required. All cultures were incubated at 25°C unless otherwise stated. Transductions were carried out using bacteriophage P22 followed by selection of non-lysogens on Green agar [91,92]. For complementation assays SL1344 was transformed with the low copy, F1-replicon-based, plasmid p*ssrAB*, kindly provided by Michael Hensel [93]. Plasmids p1437-1 and p1437-6, the P15A-replicon-based, arabinose-inducible *ssrAB+ *and *ssrAB*- (reverse orientation) derivatives were used to over-express SsrA. Antibiotics were added at the following concentrations: kanamycin (Km), 50 μg ml^-1^; carbenicillin (Cb), 100 μg ml^-1^; chloramphenicol (Cm), 25 μg ml^-1^. Media supplements were sterilised by filtration through 0.22 μm filters (Sartorius) and added to culture media. All non-aromatic amino acids (i.e alanine, cysteine, glutamate, glutamine, histidine, and serine) were added at 0.1, 1 and 10 mM. All aromatic amino acids were tested at the concentrations shown in Figures 6 and 7.

**Table 4:** Bacterial strains and plasmids.

Strain/plasmid	Relevant characteristics	Reference/source
***S. enterica *serovar Typhimurium strains**		
SL1344	WT (*rpsL hisG*) mouse virulent	[109,110]
JH3182	SL1344/*ΔSTM2779::*Km	This study
JH3183	SL1344 *ΔygaT::*Km	This study
JH3184	SL1344 *ΔybaY::*Km	This study
JH3185	SL1344 *ΔtrpE::*Km	This study
JH3186	SL1344 *ΔyhfG::*Km	This study
JH3187	SL1344 *ΔSTM0341::*Km^$^	This study
JH3188	SL1344 *Δmtr::*Km	This study
JH3189	SL1344 *ΔpspA-E::*Km	This study
JH3142	SL1344 *ΔrpoS::*Amp	Laboratory collection
JH3180	SL1344 *ssrA*::mTn*5*(Km^r^)*	This study
JH3181	SL1344 *ΔssrA*(pSsrAB)	This study
JH3179	SL1344 *Δorf319*::Cm	This study
LT2A	WT lab strain	[100]
JH4000	LT2A *Δhns*	[111]
**Plasmids**		
pKD4	Template plasmid, Km^R^	[103]
pKD46	Lambda Red helper plasmid, Amp^R^	[103]
pWSK29	p*ssrAB*	[58]
p1437-1	*bla pBAD-ssrAB*^+^	K. Tedin
p1437-6	*bla pBAD-ssrAB*^-^	K. Tedin

* The *ssrA*::mTn*5 *mutation [111] was transduced to SL1344 from strain P3F4 (12023 *ssrA*::mTn*5*).

^$ ^It should be noted that the polar mutation in STM0341 could affect expression of the downstream STM0342 gene; both of these genes are highly down-regulated during biofilm growth. Therefore, the putative periplasmic protein encoded by STM0342 may play a role in the phenotypes associated with strain JH3187.

### Biofilm formation on glass and polystyrene

The batch biofilm system used for *in situ *analysis of bacterial attachment to glass was designed at the Environmental Microbiology Research Group at Exeter [94]. This system provides a way of monitoring biofilm formation on a hydrophilic glass surface under flowing conditions. Overnight cultures of *S*. Typhimurium SL1344 were grown in CFA and standardized to achieve an initial concentration of 10^6 ^CFU ml^-1 ^and injected into a stirring influent flask containing 5 L of pre-warmed (25°C) sterile CFA medium. The inoculated influent was then pumped through silicon tubing (5 mm internal diameter, Samco Silicon Products Ltd.) at 60 ml h^-1 ^using a peristaltic pump (Minipulse 3, Gilson). In this manner, the bacteria were allowed to flow through the closed system and either attach to a borosilicate glass flow cell (3 × 3 mm, Camlab), held in a heated microscope stage, or flow out into a waste reservoir. A laminar flow rate was chosen to produce minimal fluctuations in velocity, requiring the bacteria to undergo active transport in order to interact with the surface [95]. The entire system was kept at a constant temperature of 25°C and biofilm formation within the glass flow cell was imaged *in situ *without interrupting the flow for up to 72 h. Any possible backflow of media or bacteria within the system was eliminated using media breaks. Attached cells were then examined using light microscopy *(see below)*.

Biofilm formation on polystyrene is based on the ability of bacteria to attach to the wells of microtitre plates, using a modification of a previously described technique [63]. This system provides a way to monitor biofilm formation on a hydrophobic polystyrene surface under static conditions. Overnight cultures of SL1344 were standardized (see above) and inoculated in quadruplicate into polystyrene 96-well plates (Nunc) containing sterile media. The plates were incubated statically for up to 48 h and then rinsed with dH_2_0 to remove any non-adherent bacteria, dried for 1 h at 60°C and stained with crystal violet (CV, BDH). The wells were destained with 20% acetone in ethanol (99.8%) solution and the amount of CV, indicative of the binding ability of each strain, was determined at A_590 _using a Spectramax Plus (Molecular Devices) spectrophotometer. All experiments were repeated on three separate occasions. The results are reported as a mean value of absorbance and analyzed for significance (P < 0.05 or < 0.01) by the Student's two-tailed t-test.

### Microscopy, image analysis and cell staining

Microscopy and image analysis of biofilms grown in glass flow cells (see above) were performed using a DMLB video light microscope (Leica). Images of bacterial attachment on the glass surface were visualized using a COHU 4612-5000 CCD camera (COHU) connected to a Macintosh G3 computer and Scion VG-5 PCI framestore board (Scion Corporation). Area measurements were calculated using Scion Image (Scion Corporation). Biofilm accumulation was measured as percentage surface cover on the top and bottom surfaces of the glass flow cell. The coordinates of 6 specific areas on the glass flow cell were marked in order to return to the same site at each time point. Images were captured for up to 72 h of flow with each time point representing an average of 30 frames. A threshold was applied to each image and the number of black pixels in each frame were measured and the percentage of biofilm surface cover was calculated as the proportion of white pixels to the total frame and analyzed for significance (P < 0.05 or < 0.01) by the Student's two-tailed t-test. To determine cell viability of the biofilm based on membrane integrity, glass flow cells were rinsed with PBS to remove any unattached cells and stained with Live/Dead *Bac*Light (Molecular Probes) for 1 h at room temperature. After 1 h, the staining solution was drained, the flow cell filled with fresh PBS and cells were viewed using a 40× objective of a CH-2 light microscope and illuminated by a BH2-RFCA fluorescent light source using a BP405 (nm) filter block (Olympus).

### Isolation of biofilm and planktonic cells for total RNA and protein extractions

Biofilm and planktonic cells used for total RNA and protein extractions were grown in a modified batch system (Additional file 1). This system is identical to the glass flow cell system described above apart from replacing the glass flow cell with silicone rubber tubing that permits the harvesting of large amounts of biofilm biomass [32,76,96]. Our rationale for choosing a 'batch' biofilm system as opposed to a 'seeded' system was to mimic a food processing environment where bacteria attach and are then subjected to a hydrodynamic environment that lacks fresh nutrients. Overnight cultures of SL1344 grown in CFA broth were standardized to achieve an initial concentration of 10^6 ^CFU ml^-1 ^and injected into a stirring influent flask containing 5 L of pre-warmed (25°C) sterile CFA medium. The inoculated influent was then pumped through silicon tubing (5 mm internal diameter, Samco Silicon Products Ltd.) at 60 ml h^-1 ^using a peristaltic pump (Minipulse 3, Gilson). The bacteria were allowed to flow through the closed system and either attach to a vertical piece of silicon tubing (1 meter length, 16 mm internal diameter, Samco Silicon Products Ltd.) or flow out into a waste reservoir. The tubing was positioned vertically to collect biofilm cells adherent to the tubing and minimise collecting bacteria that had simply sedimented. Possible backflow of media or bacteria into the influent from the silicon tubing was eliminated using media breaks. Samples taken to determine the pH of the medium or bacterial cell counts were removed via a media port located near the effluent and influent vessel, respectively. Planktonic cells were removed after 6, 24 and 72 h of growth from the influent vessel to avoid any contamination with biofilm cells. The entire system was kept at a constant temperature of 25°C and biofilm cells were isolated after 72 h of growth. All planktonic and biofilm samples were isolated from the same experimental system and used for both RNA and protein isolation.

### Protein extraction and 2-D analysis

The silicon tubing used for isolating biofilm cells was rapidly rinsed three times in cold (4°C) PBS to remove any non-adherent cells. A sterile loop was used to detach the adherent cells from the silicon tubing into 20 ml of cold (4°C) PBS. Total protein was extracted using a previously described technique [97]. Briefly, cells were lysed in a Triton-X-100 solution containing phenolmethylsulfanofluoride (PMSF) and sonicated in 15 s bursts for 3-5 cycles (Sanyo/MSE Soniprep 150, 4 mm probe). Protein extracts were then quantified at A_280 _(Cecil Instruments Ltd., Cambridge). The first dimension was achieved by isoelectric focusing (IPGphor™) of Immobiline drystrips (pH 4-7, Amersham) containing 0.25 mg of protein from each sample over 25 h (total of 81050 Vhrs). The second dimension was run using pre-cast 10-12% ExcelGel^® ^polyacrylamide gels and SDS buffer strips (Amersham) on a Multiphor II (Pharmacia) horizontal unit with immobilised pH gradients (pH 4-7). Gels were fixed in a 10% methanol: 7% acetic acid solution for 30 m, prior to staining overnight in SYPRO^® ^Ruby protein gel stain (Molecular Probes). A detailed description of protein sample preparation and 2-D-PAGE can be downloaded from http://www.proteome.soton.ac.uk/resources.htm. Protein spots from biofilm and planktonic gels were defined as being differentially expressed using ProteomWeaver v2.1.1. software (Definiens) and removed from the gels using a 5 ml Diamond Tip (Gilson) after an Investigator ProPick spot picker (Genomic Solutions) had located the correct position on the gel. Proteins were trypsin digested in an Investigator ProGest digestion robot (Genomic Solutions) and identified by MALDI-TOF mass spectrometry (Bruker Reflex III) and peptide mass fingerprinting using an offline version of Mascot (Matrix Sciences) to search the peptide masses [98].

### Western blot analysis

Equal amounts of protein from *S*. Typhimurium biofilm and planktonic cell extracts were separated by SDS-PAGE (5-12% gradient) using a Mini Protean 3 electrophoresis system (Biorad) according to the methods of Sambrook *et al*. [90]. For immunoblotting, samples were transferred to nitrocellulose Biodyne A membranes (Pall Corporation) using a Mini Trans-Blot Electrophoretic cell (Biorad) according to the manufactures instructions. The proteins were fixed with methanol and the membranes were blocked with 5% skim milk and 1% BSA. Immobilized protein was detected using monoclonal FliC antibody (primary antibody). Anti-mouse (titre 1:2000) or anti-rabbit (1:5,000) IgG conjugated to alkaline phosphatase (AP) were used as secondary antibodies and purchased from Sigma. 5-bromo-4-chloro-3-indolyl-phosphate and nitro blue tetrazolium (BCIP/NBT) colour development substrate was used for antibody detection (Promega).

### RNA and DNA extraction and quantification

The silicon tubing used for isolating biofilm cells was rapidly rinsed three times in cold (4°C) PBS to remove any non-adherent cells and immediately transferred to ice cold PBS containing a 1/5 volume of 5% (v/v) phenol (pH 4.3), 95% (v/v) ethanol solution and left on ice for a minimum of 30 min to stabilise the mRNA [99]. Biofilm cells were removed from the tubing using a sterile loop to detach the adherent cells into 20 ml of cold (4°C) phenol ethanol solution. RNA was isolated from biofilm cells, and planktonic cells from the influent vessel, using a SV Total RNA Isolation kit (Promega). A detailed description of the RNA isolation procedure can be downloaded from http://www.ifr.bbsrc.ac.uk/safety/Microarrays/Protocols.html#RNAextraction. Chromosomal DNA was isolated from the genomic reference strain, *S*. Typhimurium LT2A [100], using a Qiagen Genomic DNA isolation kit according to the manufacturer's instructions and digested with *Eco*R1 (Promega). DNA and RNA samples were quantified at A_260 _and A_280 _using a SpectraMax Plus (Molecular Devices). The quality of RNA samples were assessed by size chromotography on a RNA 6000 Nano Chip (Agilent) using the Agilent 2100 Bioanalyzer Software according to the manufacturer's instructions.

### Fluorescent labelling and microarray hybridization

Total RNA from biofilm and planktonic samples (16 μg) was converted to cDNA and fluorescently labelled by random priming to incorporate Cy3-dCTP (Amersham) using reverse transcriptase (StrataScript, Stratagene). Labelled cDNAs were hybridized against Cy5-labelled genomic DNA for indirect comparison microarray experiments to glass slide microarrays featuring 95% of the sequenced and annotated LT2A genome in addition to internal controls [100-102]. Information on the microarrays and complete protocols for labelling, hybridisations and slide blocking can be downloaded from http://www.ifr.bbsrc.ac.uk/safety/Microarrays/default.html#Protocols.

### Microarray data analysis

Slides were scanned using a GenePix 4000A scanner (Axon Instruments, Inc.). Fluorescent spots and local background intensities were quantified using Genepix Pro 3.0 Software (Axon Instruments, Inc.). The data were filtered so that spots with a reference signal lower than the background plus two standard deviations of the background or obvious blemishes were not included in the analysis. Signal intensities were corrected by subtracting the background and the red/green (Cy5/Cy3) ratios were calculated. To compensate for unequal dye incorporation, data centring was performed by bringing the natural logarithm of the median of spots printed by the same pin to zero. Data from each microarray that passed the quality control procedures were then analyzed using Gene Spring 7.3 (Agilent). Differentially expressed genes were identified by performing a parametric (Student's t-test) test and by correcting the P-values with the Benjamini and Hochberg false discovery rate (FDR). Genes present in *S*. Typhimurium LT2 but absent from SL1344 were filtered out and are not presented in this manuscript. The expression data for biofilm and planktonic samples presented in this study represent four technical replicates from three independent biological experiments. The expression data for the biofilm samples have been deposited in the NCBI Gene Expression Omnibus http://www.ncbi.nlm.nih.gov/geo/ and are accessible through GEO Series accession number GSE17246.

### Genetic manipulations

Deletion of selected genes on the SL1344 chromosome was achieved by one-step inactivation using PCR products, as previously described [103]. Red recombinase template plasmid pKD4 was amplified using primers (Sigma-Genosys) designed with homology extensions to regions 100 bp either side of the predicted ORF of selected genes (based on the LT2 genome sequence [100]). PCR was carried out using BIO-X-ACT DNA polymerase (Bioline) with the manufacturer's recommended 1 kb amplification parameters. Linear DNA products were introduced into SL1344/pKD46 by electroporation (Gene Pulser, Biorad). Gene disruption was verified by PCR and restriction enzyme analysis (the full list of primers used for construction and verification can be found in Additional file 5B). Following verification, the mutants were P22-transduced into a clean SL1344 background and non-lysogenic bacteria were selected on Green Agar plates.

### Phenotypic analysis of mutants

Growth curves of parent strains and mutants were performed using a Microbiology Reader Bioscreen C (ThermoLabsystems). Strains were tested for growth in CFA and LB broth over a period of 24 h. Each overnight culture was standardised to achieve an initial concentration of 10^6 ^CFU ml^-1 ^and inoculated into four separate wells of a 100 well sterile honeycomb plate (ThermoLabsystems) containing fresh medium and antibiotics as appropriate. The plates were incubated at 25°C with covers and measurements based on optical density (OD_600_) were recorded every 15 min. EPS production was monitored by growth at 25°C on CFA without salt, supplemented with 0.01% Congo Red (CR) as based on a previously described method [104]. Swimming motility was assessed by stabbing colonies into plates of CFA and LB containing 0.3% wt/vol agar and incubating at 25°C and 37°C for up to 48 h [105]. Calcofluor binding was assessed by growth on CFA agar supplemented with fluorescent brightener 28 (Sigma) as described by Zogaj *et al*. [24]. RpoS activity was examined indirectly by assessing hydrogen peroxidase II (HPII) levels as described by Zambrano *et al*. [106].

### Bacterial adherence and invasion of HeLa cells

HeLa epithelial cells (European Collection of Cell Cultures (ECACC, 93021013) were grown in HEPES-buffered DMEM (Sigma) supplemented with 10% Fetal Bovine Serum at 37°C in presence of 10% CO_2_. The infection procedure was derived from the method of Steele-Mortimer *et al*., [107] as modified by Hautefort *et.al*., [108]. To assess the invasion ability of the *Salmonella *strains, bacteria that remained outside of the HeLa cells were subsequently killed by addition of HEPES-buffered DMEM containing 10% FBS and 30 ug ml^-1 ^Gentamicin for 30 min at 37°C under 10% CO_2 _atmosphere. Infected HeLa cells were washed twice in PBS (pH 7.4) and lysed for 10 min at room temperature in PBS containing 0.1% SDS. Intracellular bacterial viable counts were estimated by plating 10-fold dilutions on LB agar plates. To assess the adherence of the *Salmonella *WT and mutant strains, infection of HeLa cell monolayers was performed as described above. After 30 min invasion, HeLa cells were washed in PBS and immediately lysed in PBS containing 0.1% SDS. The total bacterial population was determined by plating dilutions of the lysate on LB agar plates. The adhering population was estimated by subtracting the intracellular viable counts obtained from the total counts. All assays were done with six biological replicates.

## List of Abbreviations

WT: wild-type; CFA: Colonising Factor Antigen; SPI2: *Salmonella *Pathogenicity Island; CV: Crystal Violet; CR: Congo Red; rdar: red, dry and rough; *trp*: tryptophan; TTSS: Type III secretion system.

## Authors' contributions

JCDH and HMLS conceived and supervised the research. SH contributed to the design of the study, performed all experiments and data analysis. SH and JCDH wrote the manuscript. All other authors played a role in designing the laboratory experiments or analysing the data. RJMB contributed to the design of the study, the SsrA overexpression experiment and the Figures. FM contributed to the protein analysis and isolation. BC was involved in the running of the protein gels. JP contributed to the biofilm formation design and to the development of the manuscript. SL contributed to the design of the microarray and assisted in transcriptomic data analysis. All authors have read and approved the final manuscript.

## Supplementary Material

Additional file 1 **Schematic of flowing batch biofilm system**. Schematic of flowing batch biofilm system used to isolate biofilm and planktonic cells for proteomic and transcriptomic analysis. The direction of flow is from left to right and the influent was agitated used a magnetic stirrer. Planktonic cells were removed from the influent vessel and biofilm cells from the vertical silicon tubing (shown in red). Click here for file

Additional file 2 **Genes up-regulated in *S*. Typhimurium biofilm compared to planktonic cells**. A list of genes which are ≥ 2-fold up-regulated (FDR 0.01) in the biofilm at 72 h compared to 72 h planktonic cells. Click here for file

Additional file 3 **Genes down-regulated in *S*. Typhimurium biofilm compared to planktonic cells**. A list of genes which are ≥ 2-fold down-regulated (FDR 0.01) in the biofilm at 72 h compared to 72 h planktonic cells. Click here for file

Additional file 4 **Proteins spots excised from the biofilm and planktonic gel and identified by MALDI-TOF mass spectrometry**. Sample identifiers highlighted in yellow represent protein data that corresponded to mRNA expression profiles from transcriptomic analysis. The *S*. Typhimurium (STM) identifier, common name (where applicable), protein predicted function, and functional category are listed for each protein. The fold expression of proteins in the biofilm compared to the planktonic gels is indicated in column G. Fourteen proteins identified from biofilm and planktonic gels were unique, meaning that a ratio of average intensity between the gels could not be determined. Unique proteins are therefore considered as either expressed only during biofilm growth or planktonic growth, respectively. Column H indicates whether the protein expression patterns were observed in one or both biological replicates. Click here for file

Additional file 5 **(A) Biofilm-regulated genes chosen for mutational analysis**. A list of the eight genes chosen for mutational analysis along with their *S*. Typhimurium gene identifier, gene name, annotation and fold change in expression. **(B). Primers used for gene deletions**. Oligonucleotide primers used to construct and confirm chromosomal gene deletions. Click here for file

Additional file 6 **Genes up-regulated in the biofilm when compared to mid-exponential and stationary phase planktonic cells**. A list of genes which are ≥ 2-fold up-regulated (FDR 0.01) in the biofilm when (72 h) compared to both mid-exponential (6 h) and stationary phase (24 h) planktonic cells. Click here for file

Additional file 7 **Growth curves of mutants and WT SL1344 in CFA medium**. Each strain was incubated at 25°C and the turbidity (A_600_) was measured every 15 m for 24 h and compared to a growth impaired control strain (i.e. a Δ*hns *null mutant [111]). The growth curve of each strain represents an average of 4 technical replicates. Click here for file
