# Supplementary material for: The transcriptional programme of Salmonella enterica serovar Typhimurium reveals a key role for tryptophan metabolism in biofilms
Source: BMC Genomics. 2009 Dec 11;10:599. doi: 10.1186/1471-2164-10-599 (PMC2805695; doi:10.1186/1471-2164-10-599)
Supplement: Additional file 1 — Schematic of flowing batch biofilm system. Schematic of flowing batch biofilm system used to isolate biofilm and planktonic cells for proteomic and transcriptomic analysis. The direction of flow is from left to right and the influent was agitated used a magnetic stirrer. Planktonic cells were removed from the influent vessel and biofilm cells from the vertical silicon tubing (shown in red). [file 1471-2164-10-599-S1.DOC]

**BIOFILM**
