# Supplementary material for: The transcriptional programme of Salmonella enterica serovar Typhimurium reveals a key role for tryptophan metabolism in biofilms
Source: BMC Genomics. 2009 Dec 11;10:599. doi: 10.1186/1471-2164-10-599 (PMC2805695; doi:10.1186/1471-2164-10-599)
Supplement: Additional file 4 — Proteins spots excised from the biofilm and planktonic gel and identified by MALDI-TOF mass spectrometry. Sample identifiers highlighted in yellow represent protein data that corresponded to mRNA expression profiles from transcriptomic analysis. The S. Typhimurium (STM) identifier, common name (where applicable), protein predicted function, and functional category are listed for each protein. The fold expression of proteins in the biofilm compared to the planktonic gels is indicated in column G. Fourteen proteins identified from biofilm and planktonic gels were unique, meaning that a ratio of average intensity between the gels could not be determined. Unique proteins are therefore considered as either expressed only during biofilm growth or planktonic growth, respectively. Column H indicates whether the protein expression patterns were observed in one or both biological replicates. [file 1471-2164-10-599-S4.DOC]

Ndk

**NUMBER OF**

**BIOFILM**

**FOLD EXPRESSION WHEN**

**BIOLOGICAL REPLICATES**

**SAMPLE IDENTIFIER**

**STM IDENTIFIER**

**COMMON NAME**

**FUNCTION**

**FUNCTIONAL GROUP**

**COMPARED TO PLANKTONIC**

**OBSERVED IN (OUT OF 2)**

6953

STM 4152

RplL

**50S ribosomal protein L7/L12 (L8).**

Translation

+ 6.9

2

2965

STM 2433

Crr

**PTS system, glucose-specific IIA component (EIIA-GLC) (Glucose- permease IIA component) (Phosphotra**

Carbohydrate metabolism

-1.4

2

3413

STM 1184

FlgL

**flagellar biosynthesis; hook filament junction protein**

Cell motility

+ 44.0

2

3399

STM 1731

n/a

**putative catalase**

Inorganic ion transport & metabolism

+ 10.1

2

8562

STM 1183

FlgK

**flagellar biosynthesis; hook-filament junction protein (HAP1)**

Cell motility

+ 3.3

2

3708

STM 0598

EntA

**2,3-dihydro-2,3-dihydroxybenzoate dehydrogenase**

Metabolism of cofactors & vitamins

UNIQUE TO BIOFILM

2

3075

STM 1796

TreA

**trehalase, periplasmic**

Metabolism of complex carbohydrates

UNIQUE TO BIOFILM

2

3097

STM 0776

GalE

**UDP-galactose-4-epimerase**

Carbohydrate metabolism

+ 2.9

2

8233

STM 1960

FliD

**flagellar-hook assoc protein 2 (HAP2), filament cap protein, enables assembly**

Cell motility

UNIQUE TO BIOFILM

2

3415

STM 2199

CirA

**Outer membrane porin, receptor for Colicin A, requires TonB**

Unassigned

UNIQUE TO BIOFILM

2

3224

STM3065

YggE

**Putative periplasmic immunogenic protein**

Unassigned

+ 3.7

2

3918

STM 0837

YbiS

**Putative periplasmic protein (exported)**

Unassigned

- 12.7

1

8744

STM 2355

ArgT

**ABC superfamily; lysine/arginine/orthinine transport protein**

Membrane transport

+ 4.9

2

3299

STM 1729

YciF

**putative cytoplasmic protein**

Unassigned

UNIQUE TO BIOFILM

2

3651

STM 2526

Ndk

**nucleoside diphosphate kinase**

Nucleoide metabolism

+ 56.4

2

2843

STM 2526

Ndk

**nucleoside diphosphate kinase**

Nucleoide metabolism

+ 13.2

2

3325

STM 0435

YajQ

**putative cytoplasmic protein**

Unassigned

UNIQUE TO BIOFILM

2

8664

STM 3106

AnsB

**periplasmic L-asparaginase II**

Amino acid metabolism

+ 2.9

2

2986

STM 4229

MalE

**ABC superfamily; maltose transport protein, substrate recognition for transport and chemotaxsis**

Membrane transport

+ 9.9

1

9188

STM 3867

AtpA

**membrane-bound ATP synthase, F1 sector, alpha-subunit**

Energy metabolism

+ 3.2

2

2952

STM 3630

DppA

**ABC superfamily dipeptide transport protein**

Membrane transport

+ 10.7

2

3404

STM 2777

IroN

**TonB-dependent siderophore receptor protein**

Inorganic ion transport/ Island

+ 4.1

1

3124

STM 2555

GlyA

**serine hydroxymethyltransferase**

Amino acid metabolism

+ 13.7

2

8677

STM2489

DapA

**dihydrodipicolinate synthase**

Amino acid metabolism

+ 2.4

2

2867

STM 0739

SucD

**succinyl-CoA synthetase, alpha subunit**

Carbohydrate metabolism/ TCA cycle

+ 2.3

2

8988

STM 3359

Mdh

**malate dehydrogenase**

Carbohydrate metabolism

+ 3.8

1

**PLANKTONIC**

2965

STM 2433

Crr

**PTS family, glucose specific IIA component**

Carbohydrate metabolism

- 1.4

2

8790

STM 1784

YchF

**putative GTP-binding protein**

Unassigned

- 11.4

2

8796

STM 0981

RpsA

**30S ribosomal subunit protein S1**

Translation

UNIQUE TO PLANKTONIC

2

8772

pSLT 048

TlpA

**alpha-helical coiled coil protein**

Unassigned

UNIQUE TO PLANKTONIC

2

8672

STM 0012

DnaK

**chaperone Hsp70 in DNA biosynthesis/call division**

Unassigned

- 17.8

2

3757

STM 2456

EutL

**putative carboxysome structural protein, ethanolamine utilisation**

Putative structures

- 36.7

2

3775

STM 4561

OsmY

**Rpos-dependent stationary phase gene, hyperosmotically inducible periplasmic protein**

- 2.8

2

8648

STM 0608

AhpC

**alkyl hydroperoxidase reductase, detoxification of hydroperoxides**

Unassigned

- 2.3

2

3055

STM 4081

TpiA

**triosephosphate isomerase**

Carbohydrate metabolism

UNIQUE TO PLANKTONIC

2

3749

STM 1378

PykF

**pyruvate kinase, fructose stimulated**

Carbohydrate metabolism

UNIQUE TO PLANKTONIC

2

8474

STM 3359

Mdh

**malate dehydrogenase**

Carbohydrate metabolism

- 2.0

1

8776

STM 4081

TpiA

**triosephosphate isomerase**

Carbohydrate metabolism

UNIQUE TO PLANKTONIC

2

3918

STM 0837

YbiS

**putative periplasmic protein**

Unassigned

- 12.7

1

3321

STM 4351

**putative arginine-binding periplasmic protein**

Amino acid transport and metabolism

- 5.6

1

2978

STM 2285

GlpB

**sn-glycerol-3-phosphate dehydrogenase (anaerobic) membrane anchor subunit**

Metabolism of complex lipids

- 4.5

2

3818

STM 4512

IadA

**isoaspartyl dipeptidase**

Unassigned

UNIQUE TO PLANKTONIC

2

9358

STM 0614

YbdQ

**putative universal stress protein UspA & related nucleotide binding protein**

Unassigned

UNIQUE TO PLANKTONIC

1

8548

STM 3359

Mdh

**malate dehydrogenase**

Carbohydrate metabolism

UNIQUE TO PLANKTONIC

2

Proteins spots exised from the biofilm and planktonic gel and identified by MALDI-TOF mass spectrophotometry (n= 2). Sample identifiers highlighted in yellow represent

protein data that corresponded to mRNA expression profiles from transcriptomic analysis. The S. Typhimurium (STM) identifier, common name (where applicable), protein

predicted function, and functional category are listed for each protein. The fold expression of proteins in the biofilm compared to the planktonic gels is indicated in column G.

Fourteen proteins identified from biofilm and planktonic gels were unique, where a ratio of average intensity between the gels could not be determined. Unique proteins are

therefore considered as either expressed only during biofilm growth or planktonic growth, respectively. Column H indicates whether the protein expression patterns were

observed in one or both biological replicates.
