# Supplementary material for: The transcriptional programme of Salmonella enterica serovar Typhimurium reveals a key role for tryptophan metabolism in biofilms
Source: BMC Genomics. 2009 Dec 11;10:599. doi: 10.1186/1471-2164-10-599 (PMC2805695; doi:10.1186/1471-2164-10-599)
Supplement: Additional file 5 — (A) Biofilm-regulated genes chosen for mutational analysis. A list of the eight genes chosen for mutational analysis along with their S. Typhimurium gene identifier, gene name, annotation and fold change in expression. (B). Primers used for gene deletions. Oligonucleotide primers used to construct and confirm chromosomal gene deletions. [file 1471-2164-10-599-S5.DOC]

**(A)**

| ***S*. Typhimurium gene identifier** | **Gene name** | **Annotation^a^** | **Fold-change of gene expression in biofilm** |
| --- | --- | --- | --- |
| STM2789 | *ygaT* | Putative cytoplasmic protein | + 35.2 |
| STM3279 | *mtr* | Tryptophan-specific transport protein | + 23.3 |
| STM0465 | *ybaY* | Putative glycoprotein/ polysaccharide metabolism | + 7.9 |
| STM1723 | *trpE* | Anthranilate synthase component I | + 8.0 |
| STM3471 | *yhfG* | Putative cytoplasmic protein | + 4.6 |
| STM1686-1691 | *pspABCDEF* | Putative phage shock proteins and transcriptional activator (*pspF*) | + 2.5 (*pspB*) |
| STM0341 |  | Putative inner membrane protein | - 38.5 |
| STM2779 |  | Putative inner membrane protein | -100.0 |

a. Annotation is shown for *S*. Typhimurium LT2 genes (based on Colibase source, colibase.bham.ac.uk, accessed Oct, 2009) and fold-change in gene expression based on transcriptomic analysis of 72 h flowing biofilms compared with planktonic cultures.

**(B)**

| **Constructing chromosomal gene deletions:** | | | | | | | | |  |  |  |  |  |  |  |  |  |  |  |
| --- | --- | --- | --- | --- | --- | --- | --- | --- | --- | --- | --- | --- | --- | --- | --- | --- | --- | --- | --- |
|  |  | |  | |  |  | |  |  |  |  |  |  |  |  |  |  |  |  |
| Forward |  | |  | |  |  | |  |  |  |  |  |  |  |  |  |  |  |  |
| Reverse |  | |  | |  |  | |  |  |  |  |  |  |  |  |  |  |  |  |
|  |  | |  | |  |  | |  |  |  |  |  |  |  |  |  |  |  |  |
|  | **STM2779** | |  | |  |  | |  |  |  |  |  |  |  |  |  |  |  |  |
|  | CATTCCTATTTTCTCAAGTAGAACTTCCAACTAAAGCGAAGTGTAGGCTGGAGCTGCTTC | | | | | | | | | |  |  |  |  |  |  |  |  |  |
|  | CCTGGTAATATTTATCAGGCGCTTTGCAGACAGAAAACCACATATGAATATCCTCCTTA | | | | | | | | | |  |  |  |  |  |  |  |  |  |
|  |  | |  | |  |  | |  |  |  |  |  |  |  |  |  |  |  |  |
|  | **STM2789** | |  | |  |  | |  |  |  |  |  |  |  |  |  |  |  |  |
|  | ATGAGCATCGCCATGTCTCCCTGACCATCTACCGAGAGGAGTGTAGGCTGGAGCTGCTTC | | | | | | | | | |  |  |  |  |  |  |  |  |  |
|  | CACAAAATCATACATCCGCGTAATTCCTTCGCCGTGGCGCCATATGAATATCCTCCTTA | | | | | | | | | |  |  |  |  |  |  |  |  |  |
|  |  | |  | |  |  | |  |  |  |  |  |  |  |  |  |  |  |  |
|  | **STM0465** | |  | |  |  | |  |  |  |  |  |  |  |  |  |  |  |  |
|  | GGGAAAGGCCTGCTGGTGCATTGATGATAAGGAGAATTGAGTGTAGGCTGGAGCTGCTTC | | | | | | | | | |  |  |  |  |  |  |  |  |  |
|  | TGTATTAACCCTCTCCGTAACGGAGAGGGTTGGCGCAGGACATATGAATATCCTCCTTA | | | | | | | | | |  |  |  |  |  |  |  |  |  |
|  |  | |  | |  |  | |  |  |  |  |  |  |  |  |  |  |  |  |
|  | **STM1723** | |  | |  |  | |  |  |  |  |  |  |  |  |  |  |  |  |
|  | TTAAGCGGGCTTTTTTTTGAACAAAATAATGAGAATAACCGTGTAGGCTGGAGCTGCTTC | | | | | | | | | |  |  |  |  |  |  |  |  |  |
|  | GTAAACGAGTCGATGTTATCGAGCAGCAGAATATCAGCCACATATGAATATCCTCCTTA | | | | | | | | | |  |  |  |  |  |  |  |  |  |
|  |  | |  | |  |  | |  |  |  |  |  |  |  |  |  |  |  |  |
|  | **STM3471** | |  | |  |  | |  |  |  |  |  |  |  |  |  |  |  |  |
|  | TATACTTGTGGTCAAGCACAGGCATATTCAGGGAGGCCAGGTGTAGGCTGGAGCTGCTTC | | | | | | | | | |  |  |  |  |  |  |  |  |  |
|  | CAGCCCCGGATAGAGGTACGGATCGCGTCCTTCGCCAAATCATATGAATATCCTCCTTA | | | | | | | | | |  |  |  |  |  |  |  |  |  |
|  |  | |  | |  |  | |  |  |  |  |  |  |  |  |  |  |  |  |
|  | **STM0341** | |  | |  |  | |  |  |  |  |  |  |  |  |  |  |  |  |
|  | GGGCTGAATTCTTTTTTGAGCATGATAATATGTCGTCTGAGTGTAGGCTGGAGCTGCTTC | | | | | | | | | |  |  |  |  |  |  |  |  |  |
|  | GATCCGATTGCGACAATGGCGATAAATATTACAAATATAACATATGAATATCCTCCTTA | | | | | | | | | |  |  |  |  |  |  |  |  |  |
|  |  | |  | |  |  | |  |  |  |  |  |  |  |  |  |  |  |  |
|  | **STM3279** | |  | |  |  | |  |  |  |  |  |  |  |  |  |  |  |  |
|  | GAGAGATAGTCACACTATAAATGACTGGAGAGAGAGCTGAGTGTAGGCTGGAGCTGCTTC | | | | | | | | | |  |  |  |  |  |  |  |  |  |
|  | AAATATTGCCGGATGAAAACAGCTCATCCGGCTCGTTCAACATATGAATATCCTCCTTA | | | | | | | | | |  |  |  |  |  |  |  |  |  |
|  |  |  | | |  |  | |  |  |  |  |  |  |  |  |  |  |  |  |
|  | **STM1686-1691** |  | | |  |  | |  |  |  |  |  |  |  |  |  |  |  |  |
|  | TAAGCGCCGCGGTTAGTATGAGTATTGAGCAATAGATTATGTGTAGGCTGGAGCTGCTTC | | | | | | | | | |  |  |  |  |  |  |  |  |  |
|  | GATAACTGGCGTAAATGTCGTCAATTAGCCGGAGAAATAGCATATGAATATCCTCCTTA | | | | | | | | | |  |  |  |  |  |  |  |  |  |
|  |  |  | | |  | |  |  |  |  |  |  |  |  |  |  |  |  |  |
| **Confirming chromosomal gene deletions:** | | | | | | |  |  |  |  |  |  |  |  |  |  |  |  |  |
|  |  | | |  |  | |  |  |  |  |  |  |  |  |  |  |  |  |  |
|  |  | | |  |  | |  |  |  |  |  |  |  |  |  |  |  |  |  |
|  | **STM2779** | | |  |  | |  |  |  |  |  |  |  |  |  |  |  |  |  |
|  | ACGGCGAACCATTCTTACAG | | | | | |  |  |  |  |  |  |  |  |  |  |  |  |  |
|  | CAGGAGTATGCGACGGTAAA | | | | | |  |  |  |  |  |  |  |  |  |  |  |  |  |
|  |  | | |  |  | |  |  |  |  |  |  |  |  |  |  |  |  |  |
|  | **STM2789** | | |  |  | |  |  |  |  |  |  |  |  |  |  |  |  |  |
|  | ATACGCGTTCCTGGAAAATG | | | | | |  |  |  |  |  |  |  |  |  |  |  |  |  |
|  | GGGGATCGGGTAGATCAAAT | | | | | |  |  |  |  |  |  |  |  |  |  |  |  |  |
|  |  | | |  |  | |  |  |  |  |  |  |  |  |  |  |  |  |  |
|  | **STM0465** | | |  |  | |  |  |  |  |  |  |  |  |  |  |  |  |  |
|  | ACCAATTCCAGGCAAAAATG | | | | | |  |  |  |  |  |  |  |  |  |  |  |  |  |
|  | ACGGCGCAATATCACTTACC | | | | | |  |  |  |  |  |  |  |  |  |  |  |  |  |
|  |  | | |  |  | |  |  |  |  |  |  |  |  |  |  |  |  |  |
|  | **STM1723** | | |  |  | |  |  |  |  |  |  |  |  |  |  |  |  |  |
|  | TCTCCCAGACCGTTGAAATC | | | | | |  |  |  |  |  |  |  |  |  |  |  |  |  |
|  | CAGTGTTTGCGCCTGATAGA | | | | | |  |  |  |  |  |  |  |  |  |  |  |  |  |
|  |  | | |  |  | |  |  |  |  |  |  |  |  |  |  |  |  |  |
|  | **STM3471** | | |  |  | |  |  |  |  |  |  |  |  |  |  |  |  |  |
|  | ATGAATCCCCAGCCGATTAC | | | | | |  |  |  |  |  |  |  |  |  |  |  |  |  |
|  | GATGTCGGGCCTTATCAGAA | | | | | |  |  |  |  |  |  |  |  |  |  |  |  |  |
|  |  | | |  |  | |  |  |  |  |  |  |  |  |  |  |  |  |  |
|  | **STM0341** | | |  |  | |  |  |  |  |  |  |  |  |  |  |  |  |  |
|  | TTCCTGAGTTTGCGTTAAGGT | | | | | |  |  |  |  |  |  |  |  |  |  |  |  |  |
|  | TCAAGCGCTATTATCGATCC | | | | | |  |  |  |  |  |  |  |  |  |  |  |  |  |
|  |  | | |  |  | |  |  |  |  |  |  |  |  |  |  |  |  |  |
|  | **STM3279** | | |  |  | |  |  |  |  |  |  |  |  |  |  |  |  |  |
|  | AATTTGACGCCAATGGTAGC | | | | | |  |  |  |  |  |  |  |  |  |  |  |  |  |
|  | TGGCAACATTAAGCTGTTCG | | | | | |  |  |  |  |  |  |  |  |  |  |  |  |  |
|  |  |  | | |  | |  |  |  |  |  |  |  |  |  |  |  |  |  |
|  | **STM1686-1691** |  | | |  | |  |  |  |  |  |  |  |  |  |  |  |  |  |
|  | AGCGCGTTGAATTCAGTTTT | | | | | |  |  |  |  |  |  |  |  |  |  |  |  |  |
|  | AGGTGAAAACGGTACGTTGC | | | | | |  |  |  |  |  |  |  |  |  |  |  |  |  |
