# Supplementary material for: The transcriptional programme of Salmonella enterica serovar Typhimurium reveals a key role for tryptophan metabolism in biofilms
Source: BMC Genomics. 2009 Dec 11;10:599. doi: 10.1186/1471-2164-10-599 (PMC2805695; doi:10.1186/1471-2164-10-599)
Supplement: Supplementary file 6 [file 1471-2164-10-599-S7.DOC]

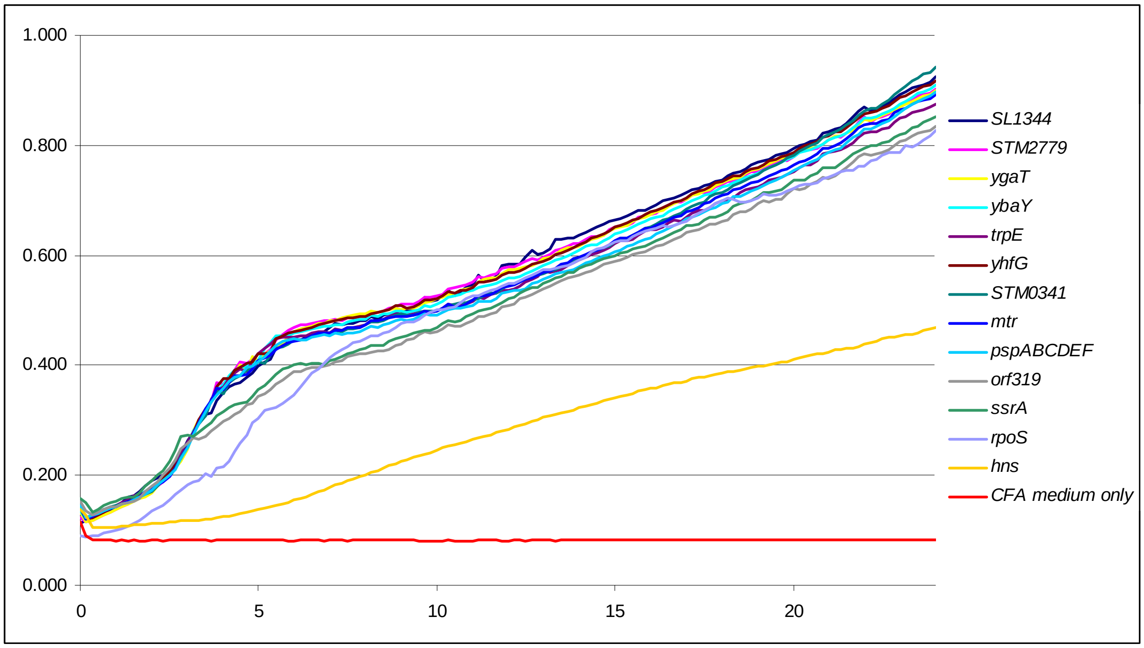

1.000
0.800
0.600
0.400
0.200
0.000
0
5
10
15
20
SL1344
STM2779
ygaT
ybaY
trpE
yhfG
STM0341
mtr
pspABCDEF
orf319
ssrA
rpoS
hns
CFA medium only
